# Harnessing environmental DNA to reveal biogeographical patterns of non-indigenous species for improved co-governance of the marine environment in Aotearoa New Zealand

**DOI:** 10.1038/s41598-023-44258-5

**Published:** 2023-10-10

**Authors:** Ulla von Ammon, Paula Casanovas, Xavier Pochon, Martin Zirngibl, Kaeden Leonard, Aless Smith, Juliane Chetham, Dave Milner, Anastasija Zaiko

**Affiliations:** 1 Cawthron Institute, Nelson, 7010 New Zealand; 2 Institute of Marine Science, University of Auckland, Auckland, 6011 New Zealand; 3 Northland Regional Council, Whangārei, 9021 New Zealand; 4 Patuharakeke Te Iwi Trust Board, Takahiwai, 0171 New Zealand; 5 Sequench Ltd, Nelson, 7010 New Zealand

**Keywords:** Molecular biology, Invasive species

## Abstract

Aotearoa New Zealand’s Northern region is a major gateway for the incursion and establishment of non-indigenous species (NIS) populations due to high numbers of recreational and commercial vessels. This region also holds a unique marine ecosystem, home to many *taonga* (treasured) species of cultural and economic importance. Regular surveillance, eradication plans and public information sharing are undertaken by local communities and governmental organizations to protect these ecosystems from the impact of NIS. Recently, considerable investments went into environmental DNA (eDNA) research, a promising approach for the early detection of NIS for complementing existing biosecurity systems. We applied eDNA metabarcoding for elucidating bioregional patterns of NIS distributions across a gradient from harbors (NIS hotspots) to open seas (spreading areas). Samples were collected during a research cruise sailing across three Aotearoa New Zealand harbors, Waitematā, Whangārei and Pēwhairangi (Bay of Islands), and their adjacent coastal waters. The small-ribosomal subunit (18S rRNA) and mitochondrial cytochrome c oxidase I (COI) genes were screened using the online Pest Alert Tool for automated detection of putative NIS sequences. Using a probabilistic modelling approach, location-dependent occupancies of NIS were investigated and related to the current information on species distribution from biosecurity surveillance programs. This study was collaboratively designed with Māori partners to initiate a model of co-governance within the existing science system.

## Introduction

Non-indigenous species (NIS) are a major threat to biodiversity, socio-cultural values, and economies worldwide^1,2^. Most marine NIS arrive either on ship hulls as biofouling or in ballast water^3,4^. Therefore, coastal locations adjacent to busy shipping nodes, like harbors or marinas are the major gateways for NIS incursions and the hotspots of their establishing populations^5^. A prominent Aotearoa New Zealand example of an international marine pest invasion is the Mediterranean fanworm *Sabella spallanzanii*, first detected in 2008 in Port Lyttleton^6^. Since then, it has spread to several harbors outcompeting native communities and posing severe problems by overgrowing submerged infrastructures^7^. Most recent incursions that are considered a major threat to ecologically and culturally valued marine environments of Aotearoa New Zealand, are the seaweeds *Caulerpa brachypus* and *Caulerpa parvifolia*^8^ that were discovered in 2021 around Great Barrier Island (Aotea) and Great Mercury Island (Ahuahu)^9^.

Aotearoa New Zealand’s biosecurity management is undertaken by a branch of the Ministry for Primary Industries, Biosecurity New Zealand, and focuses on areas of NIS occurrences that are usually considered of elevated marine biosecurity risk and prioritized by authorities for routine NIS surveillance. In Aotearoa New Zealand, this is done through bi-annual port surveys using divers who search and eradicate NIS listed as notifiable, which are those with known negative impact on native biodiversity, economic and cultural values^10,11^. The release of the Biosecurity Strategy “Biosecurity 2025” seeks to better engage all citizens in this biosecurity process and include advanced science and technology into the surveillance workflow^12^.

This includes Aotearoa New Zealand’s Māori communities, however, authentic power sharing in the management of coastal marine environments as envisaged in Te Tiriti o Waitangi (the Treaty of Waitangi, a partnership agreement between the British crown and the Māori people) was not realized^13^ and the wellbeing of iwi and hapū (tribes and subtribes) have been compromised by the alienation and poor management of natural resources. Therefore Māori are striving to have more effective participation in environmental management and decision-making (https://www.patuharakeke.maori.nz/te-taiao-environment^14^) based on the three principles of Te Tiriti o Waitangi – Partnership, Participation and Protection. Indigenous peoples maintain a deep traditional ecological understanding and spiritual connection to the land and the biota that inhabit it. There is a collective concept of kaitiakitanga (the exercise of guardianship by the Māori people of an area in accordance with Māori customary practices or behaviors in relation to natural and physical resources, and include the ethic of stewardship^15^) over many species that are considered to be taonga (treasured), and central to Māori identity and wellbeing^16,17^. As such, Māori knowledge, resources and people need to play a key part in the management of pests and diseases, predators and the technologies used for the control of NIS.

Recent emerging technologies based on eDNA, such as metabarcoding or species-specific PCR assays, promise to alleviate early NIS detections and to support more cost-efficient management responses^18^. Using metabarcoding in combination with universal marker genes such as the ribosomal nuclear 18S rRNA and mitochondrial COI genes has the power of detecting large and fine scale shifts in whole community patterns^19^, which is vital to understanding NIS expansion ranges from their source of introduction^20^. Building up trust in such novel science technologies through successful application and good communication is key in the adoption pathway by the wider public including Māori communities^16^.

Thus, our study aimed to emulate best practices for experimental co-development with Māori project partners, taking into account the specific cultural and territorial context of their rohe (territory) and applying the eDNA biomonitoring approach (18S rRNA and COI metabarcoding) to elucidate regional spread and biogeographic distribution patterns of NIS across New Zealand’s northern marine areas.

## Methods

### Experimental co-development

This study attempted to follow responsible science and appropriate partnership practices, in which scientists from western and Māori backgrounds understand and exercise their duty of care with respect^21^. The study was co-developed with the Patuharakeke Te Iwi Trust Taiao, specifically:

- Selection of sample sites was informed by Patuharakeke Te Iwi Trust Taiao’s knowledge of NIS incursions and spreading behavior;
- Sampling was undertaken by a joint team consisting of the Northland Regional Council, the Patuharakeke Te Iwi Trust Taiao and the Cawthron Institute people;
- The manuscript was conceptualized together and written with input from both western and Māori scientists;
- Te Reo Māori terminology (e.g. for sampling sites) was implemented where it seemed appropriate to reach an international audience;
- Ongoing discussions with Patuharakeke Te Iwi Trust Taiao team and regional councils were had throughout the research process to, for example, provide guidance re data analysis, storage and presentation of the research.

### Field sampling

**Figure 1:**
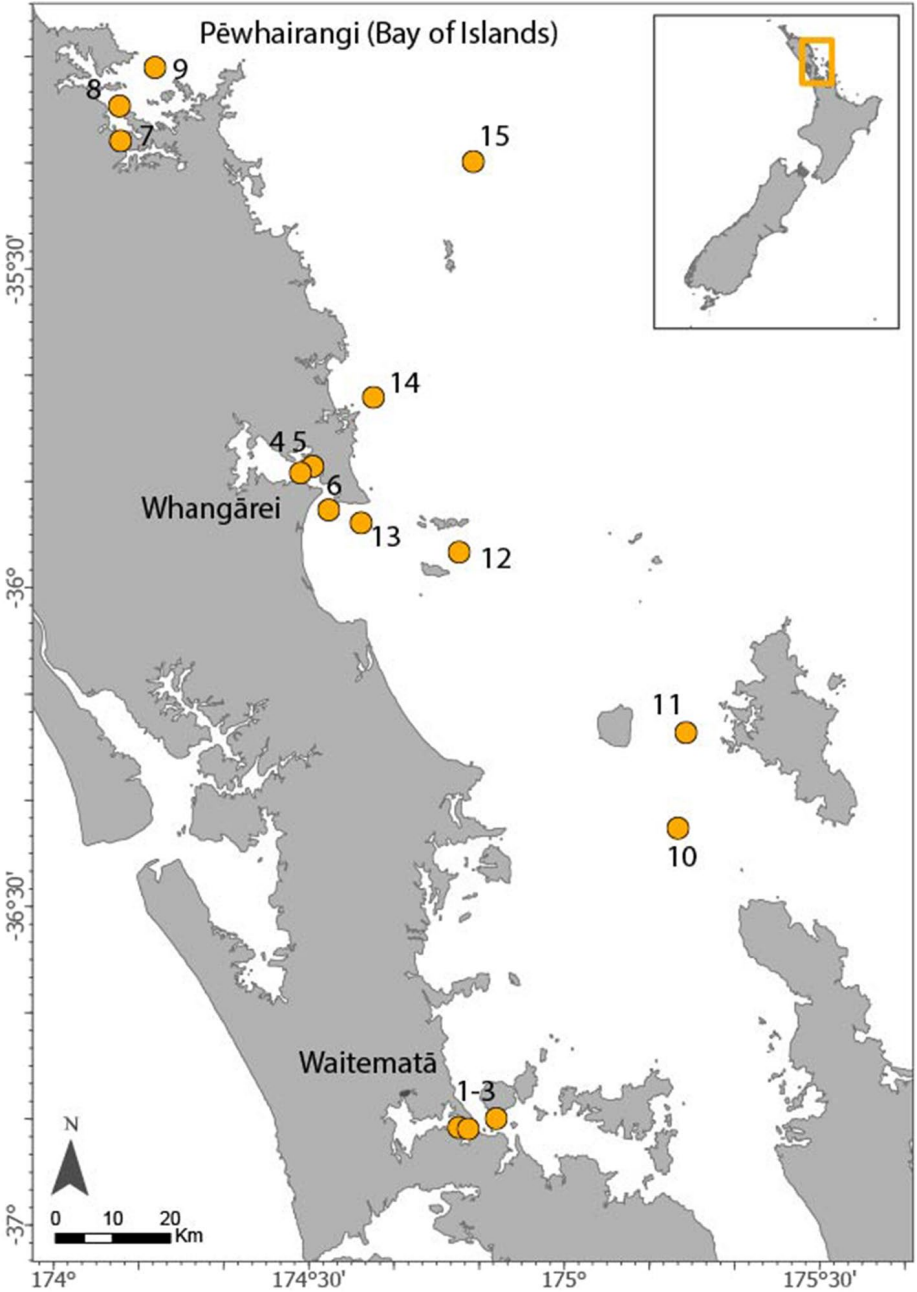
Sampling sites covering two gradients from south (Waitematā Harbor) to north (Whangārei Harbor) and from harbors (Sample site 1–9) to coastal waters sampling locations (Sample sites 10–15).

Triplicate samples per sampling site were collected in February 2020 along the eastern coast of New Zealand’s Northland region (Fig. 1), covering both a latitudinal gradient from south (Waitematā Harbor) to north (Whangārei Harbor) and a longitudinal gradient from inner to outer harbors and coastal locations (Sample sites 1–15, Table 1).

**Table 1:** Sample sites, their coordinates and description of the location.

Sampling site*	S	E	Site
1	36° 50′ 22.18	174° 47′ 28.1	Waitematā Harbor
2	36° 49′ 31.06	174°51′ 67.2	Waitematā Harbor
3	36° 50′ 22.18	174° 47′ 28.1	Waitematā Harbor
4	35° 10′ 89.85	174° 10′ 22.6	Pēwhairangi (Bay of Islands) Harbor
5	35° 14′ 56	174° 06′ 2	Pēwhairangi (Bay of Islands) Harbor
6	35° 17′ 851	174° 06′ 37	Pēwhairangi (Bay of Islands) Harbor
7	35° 52′ 335	174° 30′ 936	Whangārei Harbor
8	35° 48′ 251	174° 29′ 024	Whangārei Harbor
9	35° 48′ 89	174° 27′ 603	Whangārei Harbor
10	36° 21′ 6283	175° 12′ 173	Coastal waters, Tikapa Moana Te Moananui a Toi/ Hauraki Gulf
11	36° 12′ 6178	175° 12′ 849	Coastal waters, Tikapa Moana Te Moananui a Toi/ Hauraki Gulf
12	35° 56′ 0991	174° 46′ 113	Coastal waters, Te Akau/Bream Bay
13	35° 53′ 5066	174° 34′ 703	Coastal waters, Te Akau/Bream Bay
14	35° 41′ 664	174° 35′ 894	Coastal waters, Ngunguru Bay
15	35° 19′ 3119	174° 46′ 925	Coastal waters, Whangaruru/Rawhiti Coast

*Triplicate samples were collected at each sampling site.

Sampling was conducted onboard the SSV Robert C. Seamans operated by the Sea Education Association (SEA; Woods Hole, MA, United States) for oceanographic research and education. The Cruising Speed Net (CGW Consulting Engineers Ltd., Nelson, New Zealand) was towed behind the vessel at approximately 5 knots for 5 min. The net has a small opening of 10–20 cm diameter able to capture 2–5 m^3^ of surface (ca. 2 m depth) seawater in 5 min and retain particles larger than 20 µm in the cod-end (see von Ammon et al.^22^).

At each new station, sterile gloves were worn and all gears (i.e., sampling nets, cod-ends, collection bottles, and filtration units) were thoroughly cleaned in local seawater mixed with a 2% bleach solution for at least 10 min, then rinsed with seawater from each sampling site to avoid cross contamination from other stations.

Following each net tow, the cod-end was carefully removed and approximately 500 ml of the collected seawater was carefully poured into a sterile plastic bottle. Geographic location and time were recorded for each station at the start of each tow.

### Permissions

The Cawthron Institute holds a Special Permit with the Ministry for Primary Industries (SP822-2) that allows the taking of fish, aquatic life and seaweed for the purposes of education and investigative research.

### Filtering

All samples were processed for filtering onboard the research vessel within 1 h post collection. The collected material was carefully resuspended by shaking the plastic sample bottles, of which 200 ml of the plankton net was used for filtration. Filtration was done using sterile Whatman filters with a pore size of 5 µm (Merck KGaA, Darmstadt, Germany) as recommended in Zaiko, et al.^23^ and von Ammon, et al.^24^ and placed in a Sterifil filtration system (Merck, Darmstadt, Germany) which used a 12 V Seaflo 21 Series Water Pressure Pump 3.8LPM (MarineDeals, Auckland, New Zealand). Each filter was stored into 2 ml sterile cryotubes containing 1.5 ml of RNA Shield buffer (Zymo Research, Irvine, USA). Samples were kept at room temperature, transported to the Cawthron Institute (Nelson, New Zealand) and stored at − 80 °C until further processing.

### DNA extraction and high-throughput sequencing

In the laboratory, filters were directly transferred into BashingBead Lysis Tubes included in the DNeasy PowerSoil Pro Kit (Qiagen, Hilden, Germany) and processed on the Qiacube extraction robot. All samples were homogenized via bead beating for 2 min (1600 MiniG Spex SamplePrep, NJ, United States), and centrifuged (10,000 × *g*, 5 min, 20 °C; Eppendorf Centrifuge 5430R, Hamburg, Germany). DNA was extracted from each sample in 100 µl eluents following the manufacturer’s protocol and DNA extraction blanks (i.e., negative controls) were included for each extraction series. The quantity and quality of extracted DNA were measured using a NanoPhotometer (Implen, Munich, Germany).

Polymerase chain reactions (PCRs) were performed to amplify the ~ 400 bp V4 region of the nuclear small subunit 18S rRNA gene and a fragment of the mitochondrial COI gene. The primers used were (18S rRNA gene) Uni18SF: 5′-AGG GCA AKY CTG GTG CCA GC-3′ and Uni18SR: 5′-GRC GGT ATC TRA TCG YCT T-3′ (Zhan et al. 2013), (COI) mlCOIintF: 5′-GGW ACW GGW TGA ACW GTW TAY CCY CC-3′ and jgHCO2198: 5′-TAN ACY TCN GGR TGN CCR AAR AAY CA-3′^25^. IlluminaTM overhang adaptors were attached to the primers to allow dual-indexing as described in Kozich et al.^26^. Amplifications were undertaken on an Eppendorf Mastercycler (Eppendorf, Hamburg, Germany) in a total volume of 50 µl using 25 µl of MyFi™ taq polymerase Mix (Bioline, MA, USA), 1 µl of each primer, 20 µl of DNA-free water, and 3 µl of template DNA. Thermocycling conditions were as follows: 95 °C for 2 min, followed by 40 cycles of 95 °C for 20 s, 52 °C for 20 s, 72 °C for 20 s, and a final extension of 72 °C for 10 min. Negative (no template) controls were added. The 18S rRNA gene and COI amplification products were cleaned and normalized using SequalPrep Normalization plates (ThermoFisher Scientific) resulting in a concentration of ~ 1 ng/μl.

Amplicons were sent to Auckland Genomics (University of Auckland, Auckland, New Zealand) for library preparation on an Illumina MiSeq™ platform with MiSeq v3 Chemistry, 2 × 250 bp, 6 pM loading with 15% PhiX. Raw sequence reads are deposited in the NCBI short read archive under the accession number in the NCBI Short Read Archive under BioProject: PRJNA777358.

### Bioinformatics

Bioinformatic pipelines for both 18S rRNA gene and COI sequences were identical unless stated otherwise. Cutadapt^27^ was used to remove the primer sequences from the raw reads with a single mismatch being allowed. These trimmed sequences were subsequently processed using the DADA2 package^28^ within R^29^. The reads were truncated to 225 and 216 bp (forward and reverse reads, respectively) and filtered with a relaxed number of “expected errors” (maxEE) threshold of two (forward reads) and six (reverse reads) to retain higher read numbers. If reads did not meet this threshold, then they were discarded from further analysis. A parametric error matrix within DADA2 was constructed as per default, following sequence dereplication. Paired-end reads were merged, after singletons were discarded, with a maximum mismatch of 1 bp and a minimum overlap of 10 bp. Chimeric sequences were removed within DADA2 using the consensus option in the removeBimeraDenovo script. Amplicon sequence variants (ASVs) for the 18S rRNA gene were taxonomically classified against the PR2^30^ database using a two-step process. The DADA2 assignTaxonomy script, based on the rdp classifier^31^, was run with a bootstrap cutoff of 0.9 and then repeated at a cutoff of 0.5 for classification of higher taxonomic ranks (family and above) which had not been previously classified. For the COI dataset, anomalies in amino acid translations were detected using Multiple Alignment of Coding Sequences (MASCE^32^). This program was used to translate and align the sequences against the MIDORI reference database^33^. This was undertaken in a two-step process. Firstly, query sequences were translated using the invertebrate translation code and aligned against a subset of the MIDORI database containing only invertebrates. Any sequences with a stop codon or possessing greater than two frameshifts were then translated using the vertebrate code and aligned against a vertebrate subset of the database. Any sequences containing a stop codon or possessing greater than two frameshifts were considered as pseudogenes and removed from further analysis. After chimera (and pseudogene) checking for the COI dataset, taxonomic assignment was achieved as per Laroche et al.^34^ to reduce the number of unassigned sequences and increase taxonomic resolution using megablast from Blastn application (options: -evalue 0.001 -max target seqs 5 -task megablast -perc identity 0.8^35^); and Blastn (options: -evalue 0.001 -max target seqs 5 -task blastn) on the entire GenBank nt database. For the Blastn methods, taxonomy returned from each hit (max 5 per query sequence) was assigned to the lowest common ancestor among hits following the blastn_taxo_assignment function of the biohelper R package (https://github.com/olar785/biohelper). Parameters were based on analysis of the marine taxa in the MIDORI database. To avoid over-classification, this assignment was then corrected based on a minimum percent identity value for species level assignment (97%), and minimum percentage cover (80%) for any hit. Finally, results from the Insect classifier and the Blastn approaches were collated, and in the absence of conflicting results among methods, taxonomy was retained from the method with the highest resolution. In case of conflict, the lowest common ancestor among the different approaches was assigned. To remove possible contamination, we used the maximum sequence count for each ASV as a removal threshold^36^. This was done three times for the extraction, PCR, and sequencing controls. Thus, any ASV with fewer reads than the threshold was assumed to be from contamination and removed from further analysis. ASVs with reads higher than the threshold were reduced by the threshold number to take into account the contamination. We did not remove these ASVs completely as a possible source of contamination is among samples, and thus, complete removal would possibly remove genuine ASVs.

### Statistical analyses

All analyses were undertaken either in the ‘phyloseq v.1.40–1’ or ‘vegan v.2.6–2’ packages in R v.4.2–1^29^. Rarefaction curves on the 18S rRNA and COI datasets were generated using the ‘rarefy_even_depth’ function. Sequencing depth showed a significant effect on the biodiversity metrics, and datasets were subsampled to 3,954 and 11,214, respectively^37^.

Species richness (Observed ASVs) was calculated from the rarefied datasets and sample means compared for each site using a one-way ANOVA (analysis of variance). The Tukey–Kramer HSD (honestly significant difference) was calculated for each pair of means to determine significant differences between the factors.

To detect rare taxa as efficiently as possible for biosecurity purposes, the non-rarefied fasta reads for 18S rRNA and COI were screened with the Pest Alert Tool (https://pest-alert-tool-prod.azurewebsites.net/, Cawthron Institute, New Zealand) for ‘putative pests’, in this manuscript referred as ‘NIS’ using Minimum % sequence identity match = 99.5% and Minimum sequence length (300 bp). The received ASVs and species names were downloaded as lists and the 18S rRNA and COI metabarcoding data were filtered for these NIS ASVs for subsequent analysis. The mean NIS richness was displayed for the 18S rRNA and COI datasets per factor ‘Sampling site’ in bar plots. Combined NIS diversity on presence-absence ASV data for 18S rRNA and COI were then displayed for each Sampling Site and at species level in pie charts.

Occupancy modeling was undertaken on the non-rarefied and NIS filtered 18S rRNA and COI datasets. Two spatial covariates (factors) were considered:

- The distance of the sampling stations (Har_cov), categorical—within the harbor (1); immediately outside harbor (2) and at some distance (> 10 km) from the harbor (3).
- The latitudinal gradient (Lat_cov), categorical—south of 36° (1) and north to 36° (2). This gradient also represents proximity to the major regional sampling stations (Auckland Harbor).

We hypothesized that the NIS eDNA signal (i.e. occupancy) will attenuate from inside harbor stations to outside coastal water stations, and from south to north (i.e. with the distance from the biggest commercial port in the regions—Ports of Auckland at Waitematā Harbor). To test this hypothesis, we fit a single-season occupancy model for each NIS^20,38^, assuming constant probability of detection and occupancy dependent on harbor proximity (Har_cov) and latitude (Lat_cov) covariates. Only NIS, which were detected in more than 1 site, i.e. with occupancy at least 10% were considered for the analysis and presence-absence transformed. Occupancy models were performed within the ‘R’ statistical and programming environment^29^, using the package “unmarked”^39^. The non-parametric Scheirer-Ray-Hare test was performed to test for significant differences between occupancy estimates across the considered covariates (Har_cov and Lat_cov).

## Results

### Sequencing output

Quality filtering and denoising resulted in 1,701,353 total reads (ranging between 3954 and 119,679 reads per sample) and 13,563 ASVs for the 18S rRNA gene and 3,146,845 total reads (ranging between 11,214 and 216,588 per sample) and 16,697 ASVs for the COI gene (Table 2). Overall richness values (observed ASVs) showed significant differences between the sampling sites for the 18S rRNA and COI gene datasets (*p* < 0.05, Supplementary Table 1). The 18S rRNA marker gene reached highest diversity values in Waitematā Harbor 1–3 (Observed ASVs = 218–167) and lowest in Pēwhairangi (Bay of Islands) Harbor 7 (Observed ASVs = 73), followed by the coastal water stations 12 and 13 (Observed ASVs = 129 and 121, respectively; Supplementary Fig. 2). All COI sampling locations showed significant differences between observed ASVs to each other, but with clearly higher values in the harbor sampling sites (Observed ASVs = 659–1445) than the coastal water stations (Observed ASVs = 382—811, see Supplement Fig. 2).

**Table 2:** Total sequence reads and Amplicon Sequence Variant (ASV) counts for the 18S rRNA and COI gene datasets (not rarefied), as well as filtered reads and ASVs for non-indigenous species (NIS).

	18S rRNA	COI
Total reads	1,701,353	3,146,845
	(3954–119,679)	(11,214–216,588)
Total ASVs	13,563	16,697
NIS reads	89,160 (0–11,010)	48,380 (0–19,314)
NIS ASVs	91	93
Unique species	21	18
in > 10% of samples	9	6

### Non-indigenous species (NIS) richness and biodiversity

Screening the 18S rRNA and COI gene datasets with the Pest Alert Tool resulted in 89,160 reads (91 ASVs) and 48,380 reads (93 ASVs) that matched with marine NIS, respectively (Table 2).

**Figure 2:**
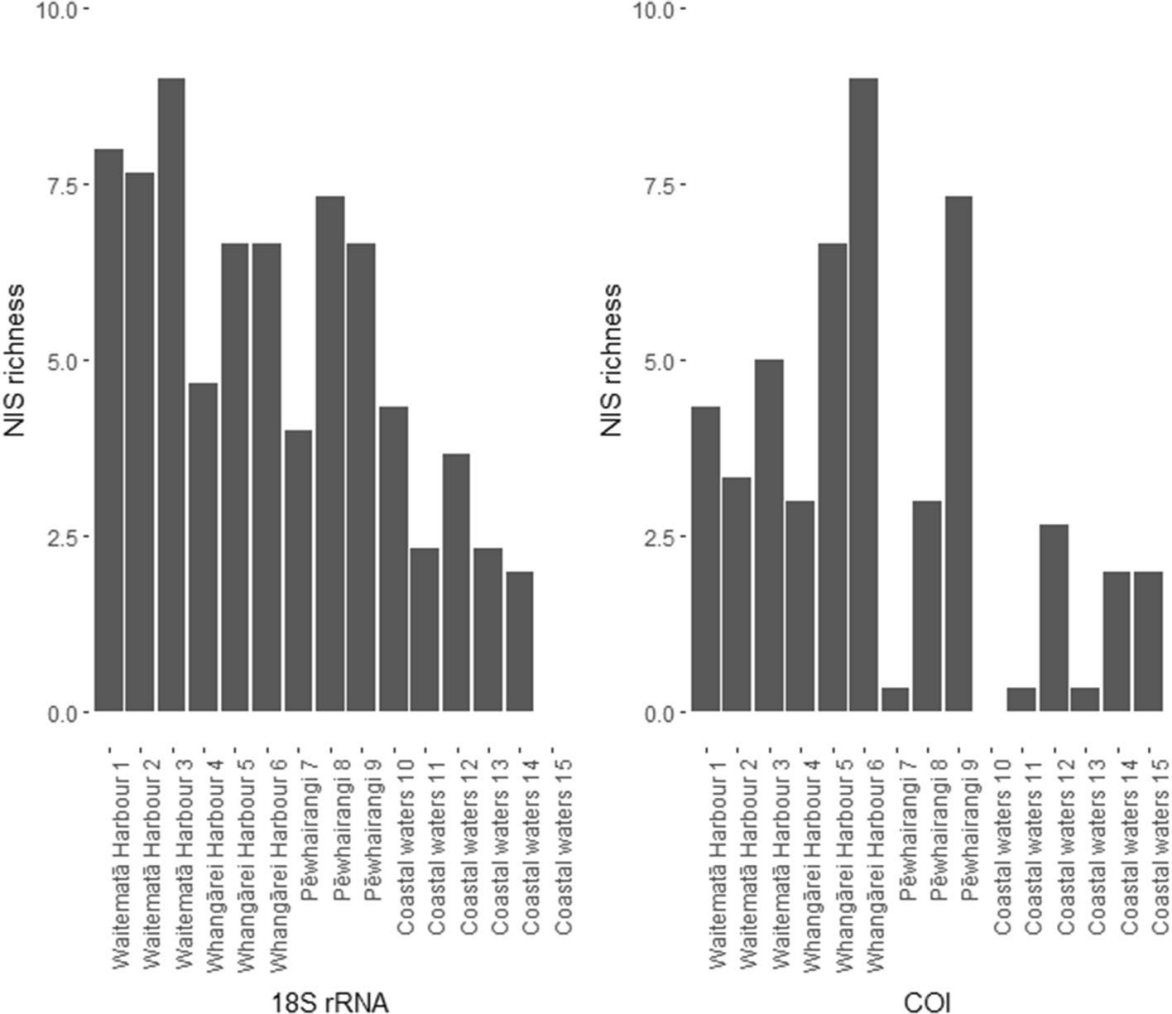
Bar plots visualizing non-indigenous species (NIS) abundance (observed Amplicon Sequence Variants [ASV]) per sampling location for the 18S rRNA and COI gene datasets.

Figure 2 displays NIS ASVs per sampling location and Table 3 shows the percentage abundance of NIS to overall ASVs per Sampling site. For the 18S rRNA gene, NIS comprised 3.50–6.38% of the ASV richness at harbor stations and 0–2.23% at the coastal stations. For COI, the NIS contribution to ASV richness was 0.79–2.15% and 0–2.09% for the harbor and coastal stations respectively (see Fig. 2 and Table 3).

**Table 3:** Total observed Amplicon Sequence Variant [ASV] counts and filtered non-indigenous species (NIS) ASV counts on unrarefied datasets and the calculated percentage (%) per sampling location and for the 18S rRNA and COI datasets.

	18S rRNA			COI		
	ASVs	NIS ASVs	%	ASVs	NIS ASVs	%
Waitematā 1	176	8	4.52	425	4	1.01
Waitematā 2	218	7	3.5	261	3	1.27
Waitematā 3	167	9	5.36	482	5	1.03
Whangārei 4	124	4	3.76	360	3	0.83
Whangārei 5	104	6	6.38	391	6	1.7
Whangārei 6	121	6	5.47	417	9	2.15
Pēwhairangi (Bay of Islands) 7	73	4	5.47	220	0	0.15
Pēwhairangi (Bay of Islands) 8	193	7	3.78	375	3	0.79
Pēwhairangi (Bay of Islands) 9	172	6	3.87	426	7	1.72
Coastal waters 10	193	4	2.23	247	0	0
Coastal waters 11	176	2	1.32	169	0	0.19
Coastal waters 12	129	3	2.83	127	2	2.09
Coastal waters 13	121	2	1.92	222	0	0.14
Coastal waters 14	216	2	0.92	270	2	0.73
Coastal waters 15	174	0	0	151	2	1.32

Replicates within locations were merged and the mean counts displayed.

**Figure 3:**
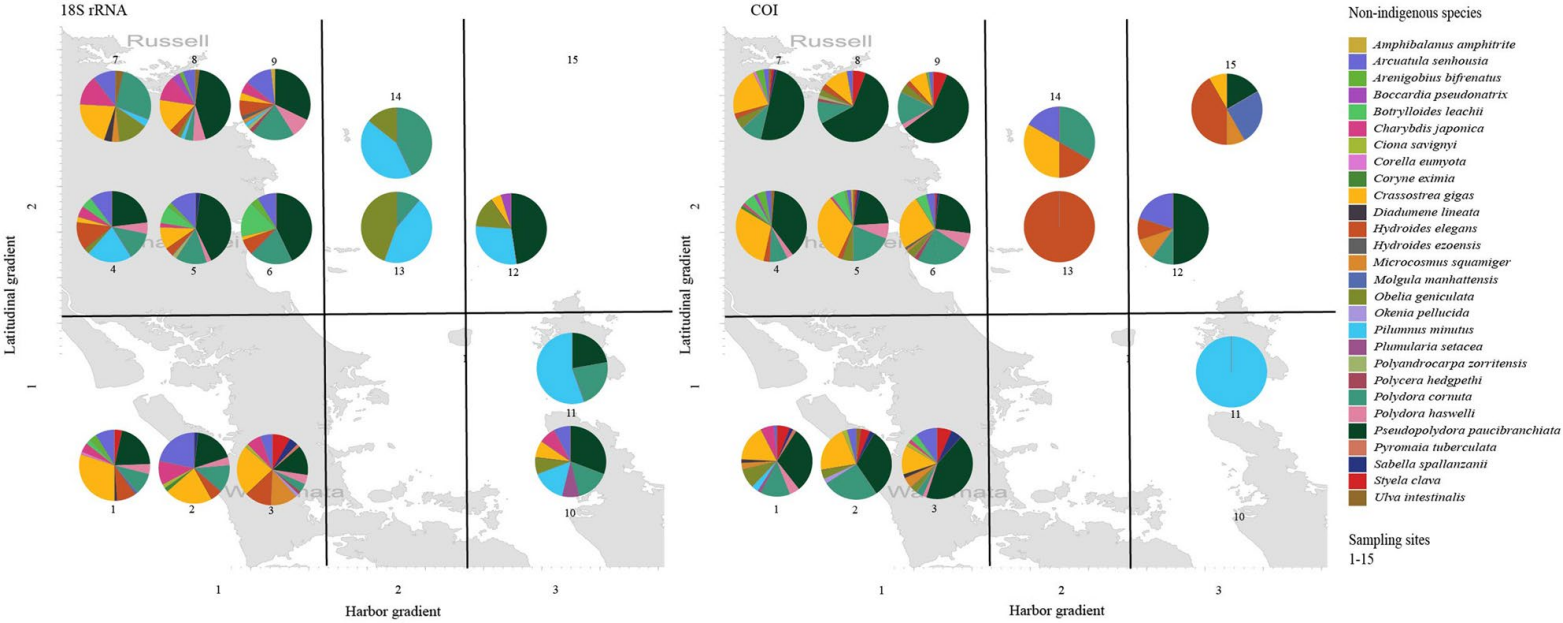
Pie charts to display non-indigenous species (NIS) abundance for the 18S rRNA gene (left side) and COI gene (right) filtered datasets. Sampling sites are divided into (I) a harbor gradient from 1–3 and (II) into a latitudinal gradient north = 1 or south = 2 of 36°.

In combination, the 18S rRNA and COI gene datasets revealed 28 unique NIS species (21 and 18 species for the 18S rRNA and COI genes, respectively, Fig. 3) with higher numbers in the harbor and close to harbor stations than the coastal water stations (Table 3). No NIS were detected at coastal stations 10 and 15 (COI and 18S rRNA genes, respectively). Most overall abundant NIS for 18S rRNA and COI genes were *Pseudopolydora paucibranchiata* (estuarine spionid polychaete)*, Crassostrea gigas* (pacific oyster) and *Polydora cornuta* (tube-building estuarine and marine polychaete)*.*

For the 18S rRNA gene, 9 unique species were present in at least 10% of the samples: *Crassostrea gigas*, *Arcuatula senhousia* (Asian date mussel), *Hydroides elegans* (tube-forming serpulid worm), *Polydora cornuta*, *Pseudopolydora paucibranchiata* (estuarine spionid polychaete), *Obelia geniculate* (hydroid), *Polydora haswelli* (mud-blister worm), *Styela clava* (clubbed tunicate) and *Pilumnus minutus* (small pea crab) and for the COI gene, 6 unique species were present in at least 10% of the samples: *Crassostrea gigas*, *Arcuatula senhousia*, *Hydroides elegans*, *Polydora cornuta*, *Pseudopolydora paucibranchiata* and *Sabella spallanzanii* (Mediterranean fanworm).

### NIS occupancy patterns across harbor proximity and latitudinal gradients

**Figure 4:**
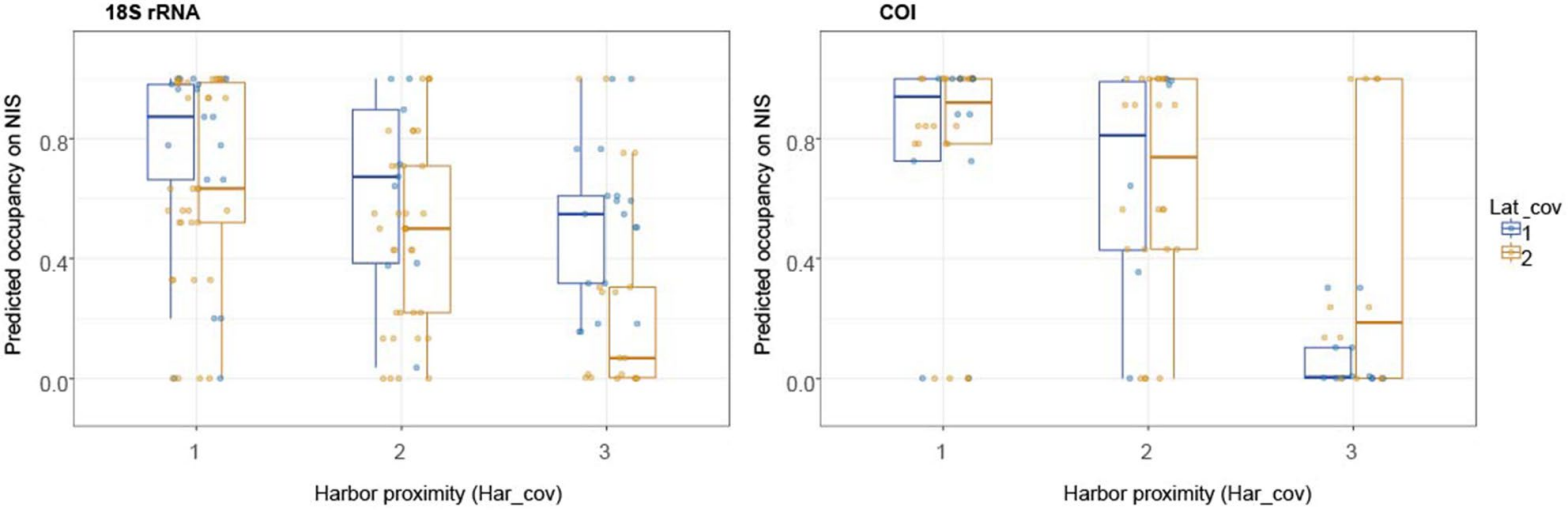
Boxplots displaying eDNA signal occupancy estimates for the non-indigenous species (NIS) contributing > 10% sequence reads in the 18S rRNA and COI gene datasets. The two spatial covariates considered were the proximity to harbors (Har_cov): within the harbor (1); immediately outside harbor (2) and at some distance (> 10 km) from the harbor (3); and latitudinal gradient (Lat_cov): south of 36° (1 [blue]) and north to 36° (2 [orange]). This gradient also represents the proximity to the major regional commercial port (Ports of Auckland at Waitematā Harbor).

The occupancy model supported our hypothesis that the NIS eDNA signal (i.e. NIS occupancy estimates) attenuates from inside harbor stations to outside coastal water stations, and from south to north (Fig. 4). Both considered co-variates (Lat_cov—grouping the sampling sites into south (1) and north (2) of 36° latitude and Har_cov—grouping samples based on proximity to the harbor) had significant effect on NIS occupancy estimates in the 18S rRNA gene dataset (*p* < 0.05, Table 4), while in the COI gene dataset, only Har_cov had a significant effect (*p* < 0.001). The overall trend observed for median occupancy values indicated a decrease of NIS signal with distance from the inner harbors and from the Ports of Auckland (Waitematā Habor stations 1–3).

**Table 4:** Scheirer-Ray-Hare non-parametric test on the individual and crossed factors Lat_cov and Har_cov.

	18S rRNA				COI			
	Df	Sum Sq	H	*p*-value	Df	Sum Sq	H	*p*-value
Lat_cov	1	7626	4.9867	*0.02*	1	323	0.4739	0.49
Har_cov	2	24,069	15.7392	*0.00*	2	12,274	18.0043	*0.00*
Lat_cov:Har_cov	2	1166	0.7627	0.68	2	561	0.8232	0.66
Residuals	129	174,480			84	46,622		

Significant *p*-values are in italic. The two spatial covariates (categorical factors) considered were the distance of the sampling sites (Har_cov), within the harbor (1); immediately outside harbor (2) and at some distance (> 10 km) from the harbor (3) and the latitudinal gradient (Lat_cov), south of 36° (1) and north to 36° (2). This gradient also represent proximity to the major regional sampling station (Waitematā Harbor).

## Discussion 

For efficient early detection of NIS and timely management responses, it is critical to understand when, where and why NIS incursions are most likely to happen, which locations are invasion hotspots and how NIS may spread into more distant environments. In this study we applied state-of-the-art molecular surveillance approaches (eDNA metabarcoding coupled with probabilistic occupancy modelling) to understand the region’s marine NIS spreading patterns and combine it with regional knowledge resources.

Waitematā Harbour has the second largest harbor in Aotearoa New Zealand and is a daily port of entry for hundreds of vessels such as container ships (21%), pleasure crafts (13%), vehicles carriers (10%), sailing vessel (10%) and passenger boats (8%). Therefore, xenodiversity is expected to be highest around this marine traffic node, attenuating with distance towards smaller harbors and marinas and even further towards more natural coastal sampling sites^5^. The present study confirmed that this pattern is observed in the eDNA signal of marine NIS. Yet, considering NIS contribution to overall biodiversity, this pattern was more obvious in the 18S rRNA gene comparing with the COI gene dataset. Usually, the 18S rRNA marker reveals a more universal and diverse fraction of plankton and includes many microorganisms^40^ whose amplification leads to higher diversities in harbor and open water sampling stations. On the other side, COI is more specific towards metazoan taxa including many biofouling organisms settling on hard substrate and artificial infrastructures^41,42^, highlighting the complementarity of using several markers for early and rare species detection^42,43^. Therefore, outside harbor stations showed expectedly lower diversity for NIS eDNA signal, likely receiving incursions via secondary spread from harbor hotspots and creating a ‘hub-and-spoke network’^44^. Recreational boating between marinas and nature sites effectively facilitate the local spread of NIS and define distribution patterns^45,46^. The crab *Pilumnus minutus*, frequenctly recorded to be transported through sea chests^47^ and the polychaete worm *Polydora cornuta* were prevalent for 18S rRNA gene in coastal water stations. For COI, again classical biofoulers such as *Hydroides elegans*, *Crassostrea gigas* and *Arcuatula senhousia* dominated^48^.

These observed patterns were also largely supported by the occupancy model outputs. Occupancy models are increasingly used to refine detection probabilities^20,49^, support decision making around study designs and aid in spatial interpretation of eDNA-based species detections^50–53^. In the present study, predicted NIS eDNA occupancy values differed significantly between the pre-determined factor groups: harbor proximity and latitudinal gradient. As hypothesized, higher NIS occupancy estimates were observed in proximity to the shipping hubs. For the 18S rRNA gene dataset, occupancy values gradually decreased towards coastal water stations and at those north of 36° latitude. The effect of the latitudinal factor was more emphasized in the 18S rRNA gene data compared with the COI gene dataset. This is likely because the 18S marker captures broader taxonomic groups, including microorganisms, while the COI gene rather depicts metazoan NIS along the coastline (*Crassostrea gigas*, *Arcuatula senhousia*, *Hydroides elegans*, *Polydora cornuta*, *Pseudopolydora paucibranchiata* and *Sabella spallanzanii*).

In total, 28 unique NIS were identified with obvious highest diversity within harbor sampling sites, demonstrating that these are actual hotspots for NIS incursions^5^. Many biofouling taxa such as the pacific oyster, *Crassostrea gigas* and spionid polychaets *Pseudopolydora paucibranchiata* and *Polydora cornuta* were dominating at these locations, and have been previously recorded in Aotearoa New Zealand through regular NIS baseline surveys conducted by Biosecurity NZ^54^. Although taxa such as *Crassostrea gigas* are considered to be aggressive invaders and are capable of transforming coastal systems and reduce habitat heterogeneity across different substrates^55^, their abundance is surveyed but eradication or management efforts are not considered. On the other hand, from a perspective of climate change and warming oceans, these resilient oysters are considered naturalized, are important kai (food) species^56^, and help restore coastal ecosystem functions. However, other detected taxa in this study are also considered priority NIS and are part of the New Zealand Register of Unwanted Organisms defined by Biosecurity New Zealand (Ministry for Primary Industries [MPI]). These include the Mediterranean fanworm *Sabella spallanzanii,* the clubbed tunicate *Styela clava* and the Asian paddle crab *Charybdis japonica*, for which control programs or integrated pest management strategies are in place^57^: For example, biannual port surveys with divers manually removing these individuals are undertaken by MPI via regular Marine High Risk Site Surveillance Surveys (MHRSS) conducted in 12 major ports by the National Institute of Water and Atmospheric Research (NIWA). For example, within these surveys, the secondary target species *Arcuatula senhousia*, *Sabella spallanzanii* and *Styela clava* were detected within the NMHRSS in 2021–22 across Waitematā and Whangārei Harbors^58^, which aligns with our eDNA findings in this study. However, *Eudistoma elongatum* appears in the NMHRSS report but not in our datasets. While reference sequences have been deployed for 18S and COI in the PAT tool for this species, naturally low eDNA shedding rates, species-specific ecology of ecology eDNA^59^ or signal loss during sampling or laboratory processing steps can be the reason for non-detection^60,61^. The other two most recent NIS that were expected in the study area, but not detected with eDNA were two algae—*Caulerpa brachypus* and *C*. *parvifolia* that so far have no reliable reference sequence available for either of the species.

Regional councils do their own surveillance on top of this effort and look at a much broader geographic area. For example, the Northland Regional Council targets about 17 major harbors and estuaries in Northland. A pathways management plan is in place, so instead of surveying for different species, at least 2000 visiting and local vessels per year are inspected and assessed for target species. However, substantial occurrence of NIS eDNA at open coast sites suggests that huge surveillance effort focused on the high-risk areas might not be enough protecting vulnerable remote ecosystems and areas of cultural importance for Māori from emerging biosecurity risks. Mana whenua in their role as kaitiaki (the Māori people who have historic and territorial rights over the land) proactively take part in mitigating these risks. Being aware that NIS may threaten significant mahinga mātaitai (seafood gathering) sites, collaboration with regional councils and other organizations in surveillance and removal operations is critical. All these combined efforts are labor-intense and require qualified, skilled people as well as specialized resources and equipment to comply with the health and safety requirements working at sea. The eDNA tools may offer a great addition to different end-user groups engaged in NIS surveillance and management and support intertwining Mātauranga Māori (Māori traditional knowledge) and western science for enhanced marine biosecurity in Aotearoa New Zealand.

The importance of establishing best-practice guidelines and quality assurance for eDNA-based biosecurity applications is crucial for implementing its nation-wide uptake for routine biosecurity practices. This has been increasingly emphasized by researchers including recognizing First Nations peoples’ ownership and stewardship^18,60,62–64^. However, it is important to acknowledge that Māori communities have reservations about molecular tools, being concerned about losing data sovereignty as the DNA sequenced of taonga (treasured) species is often sent overseas, effectively removing the Māori property rights^21^, but also concerns about the genomic sequencing of taonga (treasured) species which has implications for key values and concepts such as whakapapa (the relationship between everything and everybody in the natural world)*.* Early engagement and formation of enduring and meaningful relationships when embarking on this type of research is essential and can result in improved outcomes for all parties, the wider community and our marine environment and taonga (treasured) species. Opportunities for participation and input into the research program enables exchange and alignment of knowledge systems and Mātauranga Māori. Establishing such relationships provides a pathway for better communication around eDNA research as a method to non-invasively sample the marine ecosystem and gain biosecurity information, in this study, from just a tiny fraction (100-400 bp) of released DNA, comparable to a fingerprint from e.g. *Sabella spallanzanii.* Alongside this, Māori communities can consider the research and methods through a Mātauranga Māori framework of values to achieve their goals for management, protection and restoration of the moana.

While co-governance approaches are critical to facilitate collaboration and integration of indigenous knowledge into western science practices, it is important to avoid tokenistic efforts that do not truly value or incorporate traditional knowledge. The implementation of Vision Mātauranga into projects based on novel technologies, such as eDNA, requires careful consideration of the cultural and ethical implications of the research, as well as recognition of the diversity of indigenous perspectives and knowledge. This can be particularly challenging, as in this case, when the technologies being used are new and appear inconsistent with indigenous concepts, emphasized by a certain level of distrust in western science methodologies as a result of the impacts of colonization. As such, it is essential to engage in ongoing dialogue and consultation with indigenous knowledge holders to ensure that their perspectives and values are being incorporated meaningfully and appropriately. It requires a deep commitment to building respectful and equitable partnerships with indigenous communities, while recognizing and addressing the power imbalances that exist within the western scientific system. We are aware that this study was just a small step towards a better co-governance approach and integration of different knowledge systems, hence it is an ongoing and critical task to educate, upskill and raise the consciousness of the wider science community.

### Supplementary Information

Supplementary Information.

## Data Availability

Raw sequence reads were deposited in the NCBI Short Read Archive under BioProject PRJNA777358. Additional tables and figures are uploaded as supplementary material. Details on the bioinformatics and modeling framework can be found under the github repository https://github.com/olar785/biohelper for our taxonomy assignment as well as the details of the modeling framework under https://gitlab.com/paula_casanovas/bucket-overboard.
