# Supplementary material for: Harnessing environmental DNA to reveal biogeographical patterns of non-indigenous species for improved co-governance of the marine environment in Aotearoa New Zealand
Source: Sci Rep. 2023 Oct 10;13:17061. doi: 10.1038/s41598-023-44258-5 (PMC10564887; doi:10.1038/s41598-023-44258-5)
Supplement: Supplementary file 1 — Supplementary Information. [file 41598_2023_44258_MOESM1_ESM.docx]

**Supplement**


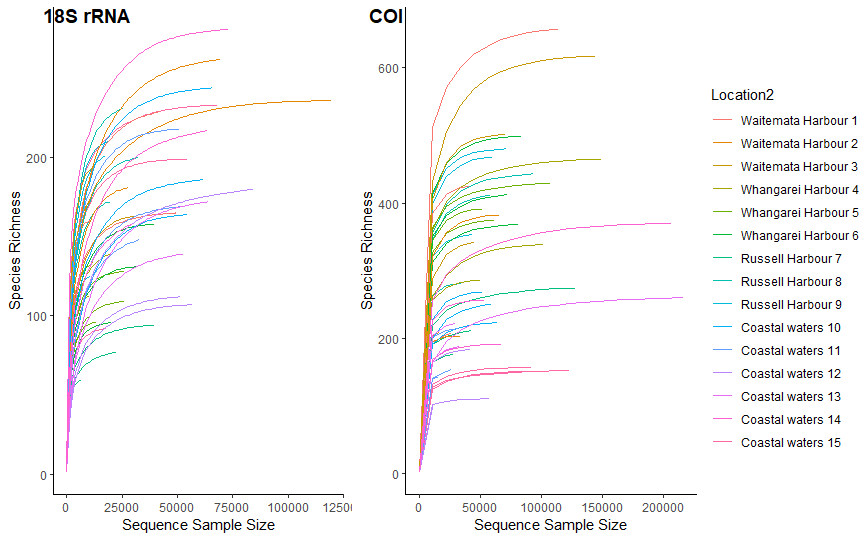


Supplementary Figure 1: Rarefaction curves on sequence read numbers for the 18S rRNA and COI datasets, colors relate to Sampling sites.

Supplementary Table 1: ANOVA for the rarefied 18S rRNA and COI datasets, displaying significant differences for the factor “Sampling Site”.

| **18S rRNA** | **Df** | **Sum Sq** | **Mean Sq** | **F value** | **Pr (>F)** |
| --- | --- | --- | --- | --- | --- |
| Sampling Site | 14 | 32588 | 2327.7 | 4.227 | ***0.000453*** |
| Residuals | 30 | 16521 | 550.7 |  |  |
| **COI** | | | | | |
| Sampling Site | 14 | 371156 | 26511 | 7.427 | ***2.36e-06*** |
| Residuals | 30 | 107087 | 3570 |  |  |


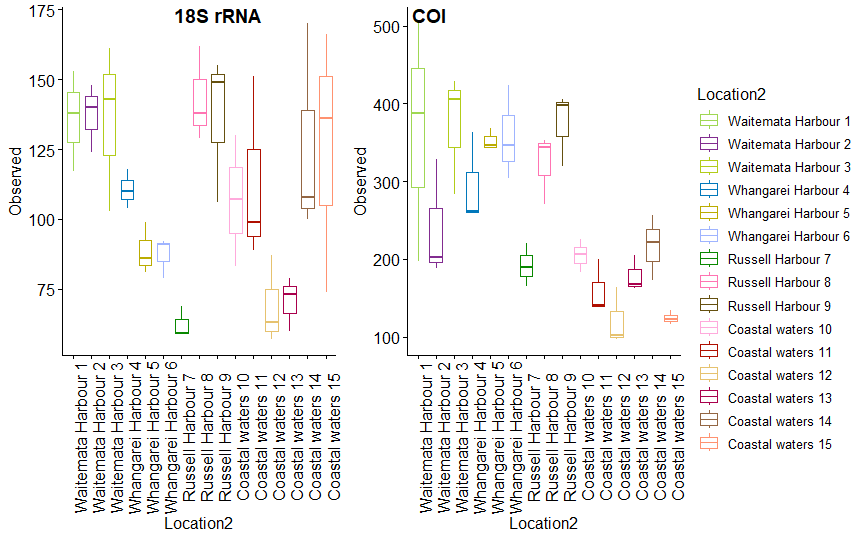


Supplementary Figure 2: Boxplots on observed ASVs per Sampling site and for the rarefied 18S rRNA and COI datasets.
